# Editorial: Nucleic acid-based therapies for cardiovascular diseases

**DOI:** 10.3389/fcvm.2024.1392073

**Published:** 2024-03-22

**Authors:** Fabio Martelli, Paras Kumar Mishra, Andrea Caporali

**Affiliations:** ^1^ Molecular Cardiology Laboratory, IRCCS Policlinico San Donato, Milan, Italy; ^2^ Department of Cellular and Integrative Physiology, University of Nebraska Medical Center, Omaha, NE, United States; ^3^ University/BHF Centre for Cardiovascular Science, University of Edinburgh, Edinburgh, United Kingdom

**Keywords:** RNA-mediated therapy, non-coding RNAs, cardiovascular disease, regenerative medicine, nucleic acid delivery

**Editorial on the Research Topic** Nucleic acid-based therapies for cardiovascular diseases

## Rationale for the research topic

The field of cardiovascular treatment discovery confronts persistent challenges in the identification of novel interventions to address the burden of heart and vascular diseases (1, 2). However, the emergence of RNA-targeted therapeutics presents a promising avenue for innovation. Beyond conventional coding mRNAs, attention has turned to the diverse array of noncoding RNA species, including microRNAs, long noncoding RNAs (lncRNAs), circular RNAs, enhancer RNAs, small nucleolar RNAs, and tRNA-derived fragments (tRFs), which hold substantial potential as therapeutic targets (3).

**Figure 1:**
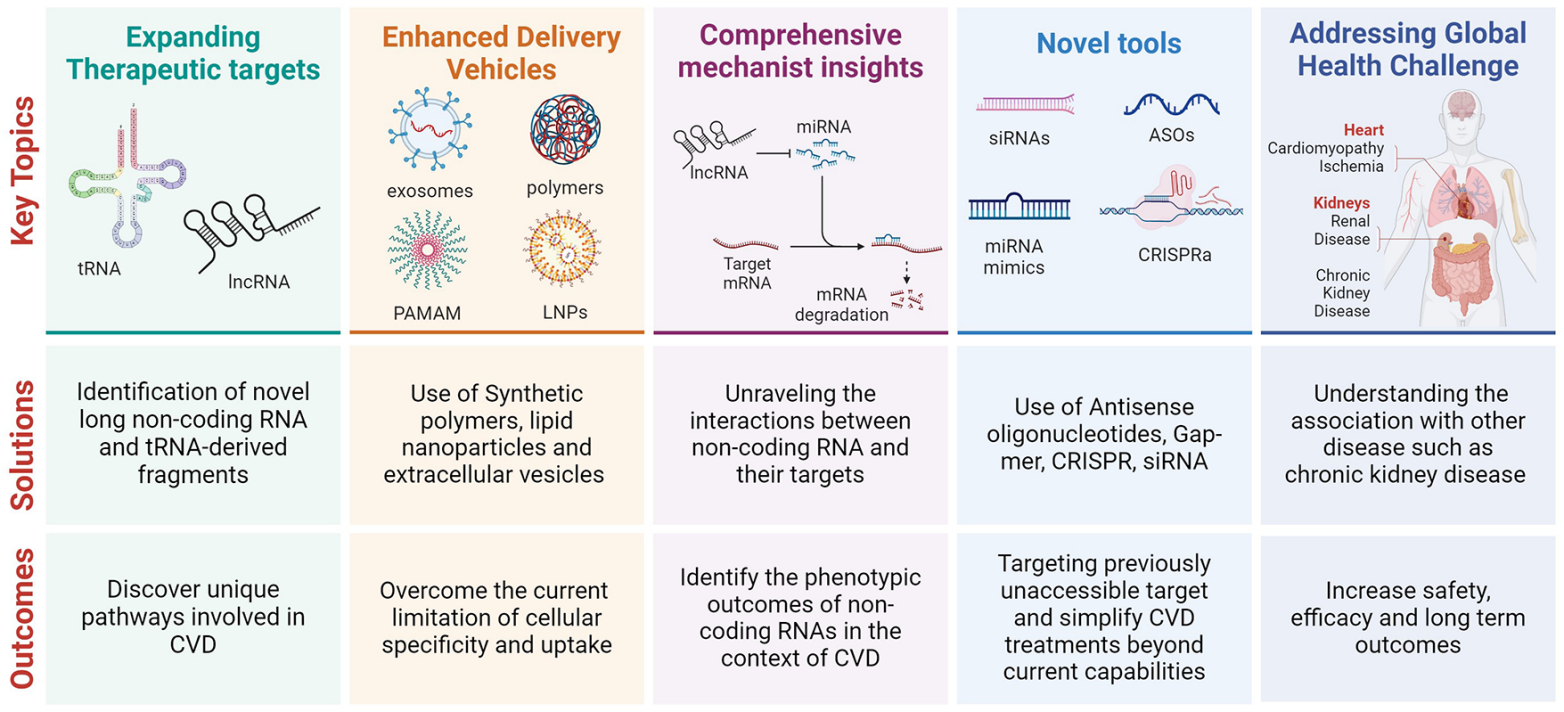
The future of nucleic acid-based therapy in cardiovascular disease.

The translation of antisense oligonucleotides and small interfering RNAs from laboratory experimentation to clinical application has been remarkable. Regulatory approvals for their clinical use underscore the viability of RNA modulation as a therapeutic strategy. Techniques such as antisense oligonucleotides, GapmeRs, synthetic pre- or anti-miRNAs, small interfering RNAs, and CRISPR technologies offer accurate and efficient tools to manipulate the transcriptome, paving the way for personalised approaches in cardiovascular medicine (4). These advancements promise to target previously inaccessible targets and simplify treatment regimens beyond current capabilities.

Notwithstanding the therapeutic potential identified in both coding and noncoding RNAs, several challenges persist in their development and optimisation. These include sequence synthesis, stability, immunogenicity, conservation, selection of efficient carriers, such as nanoparticles, and determining optimal delivery routes to ensure adequate uptake by cardiac and vascular tissues. Furthermore, additional pharmacological parameters necessitate thorough investigation.

While these are significant challenges, the prospect of overcoming them has solid ground. The successful development in a relatively short time of RNA-based COVID-19 vaccines (5), acknowledged by the 2023 Nobel Prize in Physiology or Medicine, underscores a paradigmatic shift catalysed by RNA-targeted therapeutics within the contemporary biomedical landscape. Concerted efforts in research and development hold the promise of transforming cardiovascular therapeutics through RNA-targeted interventions.

This Research Topic aimed to spotlight manuscripts that embody the fusion of robust basic research and evident translational promise for cardiovascular ailments. We have curated a collection that not only informs but also inspires the next wave of nucleic acid-based discovery and application in the realm of cardiovascular therapy.

## New findings reported

Mesenchymal stem cells (MSCs) manifest their action by the paracrine secretion. Furthermore, it has been substantially demonstrated in recent years that exosome cargo is enriched in distinct ncRNAs, making them a relevant therapeutic option in regenerative medicine (6). Chen et al. showed that exosomes from skeletal muscle cells exposed to hypoxia/reperfusion contain lncRNA KLF3-AS1 (Chen et al.) that promoted IGF-1 secretion from MSCs. Mechanistically, lncRNA KLF3-AS1 induced IGF-1 secretion from MSCs via miR-23c/STAT5B regulation. Finally, *in vivo* experiments using a rat model of Ischaemia/reperfusion showed that injection of MSCs exposed to exosomal lncRNA KLF3-AS1 promoted IGF-1, thus reducing I/R-mediated infarct size and increasing heart performance.

M1 macrophages can promote inflammation and play an essential role in regulating sympathetic remodelling after myocardial infarction. Li et al. performed RNA-seq of M0- and M1-type macrophages to identify differentially expressed lncRNAs (Li et al.). lncRNA LOC100911717 was upregulated in M1-type macrophages comp[are to M0-type macrophages. In a rat model of myocardial infarction, lncRNA LOC100911717 was upregulated in M1-type macrophages and regulates the expression of its target GAP43. This gene plays a crucial role in axonal outgrowth and synaptic plasticity. Silencing lncRNA LOC100911717 *in vivo* using Adeno-Associated viral vector (AAV) decreased sympathetic remodelling, reduced the incidence of ventricular arrhythmias and the infarcted heart area and improved cardiac function.

tRFs are a new class of ncRNAs generated by the specific cleavage of mature tRNAs or pre-tRNAs under stress conditions or normal physiological conditions. By sequencing, Rong et al. identified several tRFs during human vascular smooth muscle cells (VSMCs) phenotypic switching. Among them, tiRNA-Gly-GCC (alias tiRNA-1:33-Gly-GCC-1) has the highest abundance and deferential expression. Moreover, tiRNA-Gly-GCC is upregulated in atherosclerosis patients' vascular tissues and plasma and balloon-injured rat carotid arteries. tiRNA-Gly-GCC regulates VSMCs switching and neointimal formation after vascular injury by decreasing CBX3.

Finally, Palmer and Hunter summarise the state-of-the-art RNA-based therapies targeting the kidney in cardiovascular disease (Palmer and Hunter). The authors described the advantages of RNA-based drugs and the challenges of targeting kidney cells with siRNA. Moreover, the administration routes for kidney-targeting RNA therapies have been discussed. Finally, they proposed that megalin and cubulin could mediate exogenous RNA uptake from the kidney's tubular space. Therefore, defining the contribution of these transporters will be an essential area of study for specific RNA delivery in kidney cells.

## Future perspectives

The future direction for nucleic acid-based therapies, particularly in cardiovascular disease (CVD), suggests a paradigm shift towards more targeted and personalised treatment modalities. The insights derived from the studies on myocardial ischemia-reperfusion injury (Chen et al.), sympathetic remodelling post-myocardial infarction (Li et al.), and vascular intimal hyperplasia (Rong et al.) underscores the potential of RNA-based interventions in addressing the intricate molecular mechanisms underlying CVDs (Palmer and Hunter). A recent review elaborates on the various applications of RNA therapy (7). A few potential future directions for nucleic acid-based therapy are described below.

1. Expanding the Therapeutic Targets: Identifying and modulating specific lncRNAs and tRFs involved in pathological processes offer a novel avenue for therapeutic intervention. For instance, the modulation of lncRNA KLF3-AS1 (Chen et al.) and LOC100911717 (Li et al.), as well as tiRNA-Gly-GCC (Rong et al.), highlights the potential to target unique molecular pathways involved in myocardial injury, sympathetic remodelling, and vascular disease progression, respectively.
2. Enhanced Delivery Mechanisms: Developing sophisticated delivery vehicles, such as synthetic polymers and engineered extracellular vesicles, is crucial for the targeted delivery of RNA-based therapeutics (Palmer and Hunter). These platforms promise to overcome the current limitations of tissue specificity and cellular uptake, thereby enhancing the efficacy and safety of these treatments.
3. Integration with Regenerative Medicine: The interplay between RNA-based therapies and regenerative medicine, as illustrated by the modulation of MSCs through exosomes, opens new doors for synergistic therapies (Chen et al.). This approach not only aims at molecular intervention but also harnesses the regenerative potential of stem cells to repair and regenerate damaged tissues.
4. Comprehensive Mechanistic Insights: Future research should unravel the complex interactions between various RNA molecules, their target genes, and the consequent phenotypic outcomes. This includes understanding the regulatory networks involving lncRNAs, miRNAs, and tRFs in the context of CVD pathophysiology.
5. Clinical Translation and Safety Evaluation: As RNA-based therapies advance towards clinical application, rigorous evaluation of their safety, efficacy, and long-term outcomes in humans becomes imperative (7). This includes assessing the potential off-target effects, immune responses, and the stability of these therapies *in vivo*.
6. Addressing Global Health Challenges: Given the significant burden of CVD and its association with conditions like chronic kidney disease, RNA-based therapies could offer a strategic intervention point (Palmer and Hunter). By targeting the molecular underpinnings of kidney disease and its cardiovascular implications, these therapies could reduce the global health burden of CVD.
7. Collaborative Research and Development: The transition from bench to bedside for RNA-based therapies will require concerted efforts across academia, industry, and regulatory bodies. This collaboration is essential for accelerating the development of novel therapies, optimising delivery systems, and ensuring the broad accessibility of these treatments.

In conclusion, the future of nucleic acid-based therapies in cardiovascular disease holds great promise, with the potential to revolutionise the treatment landscape through precision medicine. By leveraging the insights from current research and focusing on the areas outlined, this promising field can move closer to realising its full therapeutic potential.

## References

[1] Heidenreich PA Bozkurt B Aguilar D Allen LA Byun JJ Colvin MM 2022 AHA/ACC/HFSA guideline for the management of heart failure: a report of the American college of cardiology/American heart association joint committee on clinical practice guidelines. Circulation. (2022) 145:e895–1032. 10.1161/CIR.0000000000001063 35363499

[2] Khakoo AY Yurgin NR Eisenberg PR Fonarow GC. Overcoming barriers to development of novel therapies for cardiovascular disease: insights from the oncology drug development experience. JACC Basic Transl Sci. (2019) 4:269–74. 10.1016/j.jacbts.2019.01.011 31061928 PMC6488739

[3] Robinson EL Baker AH Brittan M McCracken I Condorelli G Emanueli C Dissecting the transcriptome in cardiovascular disease. Cardiovasc Res. (2022) 118:1004–19. 10.1093/cvr/cvab117 33757121 PMC8930073

[4] Sheridan C. Genetic medicines aim straight for the heart. Nat Biotechnol. (2023) 41:435–7. 10.1038/s41587-023-01745-4 37016163

[5] Chavda VP Jogi G Dave S Patel BM Vineela Nalla L Koradia K. mRNA-based vaccine for COVID-19: they are new but not unknown!. Vaccines (Basel). (2023) 11(3):507. 10.3390/vaccines11030507 36992091 PMC10052021

[6] Pant T Juric M Bosnjak ZJ Dhanasekaran A. Recent insight on the non-coding RNAs in mesenchymal stem cell-derived exosomes: regulatory and therapeutic role in regenerative medicine and tissue engineering. Front Cardiovasc Med. (2021) 8:737512. 10.3389/fcvm.2021.737512 34660740 PMC8517144

[7] Kim YK. RNA therapy: rich history, various applications and unlimited future prospects. Exp Mol Med. (2022) 54:455–65. 10.1038/s12276-022-00757-5 35440755 PMC9016686

